# Facteurs de prédiction de réadmission précoce et mortalité dans l’insuffisance cardiaque dans le Service de Médecine Interne de l’Hôpital Universitaire San Carlos, Espagne

**DOI:** 10.11604/pamj.2019.34.202.17356

**Published:** 2019-12-17

**Authors:** Noel Lorenzo Villalba, Belén Chiva Ballesteros, Laura De Pedro Álvarez, Pamen Delgado Mainar, Ángel Nieto Sánchez, Javier Marco Martínez, Elpidio Calvo Manuel, Manuel Méndez Bailon

**Affiliations:** 1 Service de Médecine Interne, Diabète et Maladies Métaboliques, Hôpitaux Universitaires de Strasbourg, France; 2 Service de Médecine Interne, Hôpital Universitaire San Carlos, Madrid, Espagne

**Keywords:** Hospitalisations, insuffisance cardiaque, réadmissions, facteurs de prédiction, pluripathologie, polypharmacie, Hospitalizations, cardiac insufficiency, readmissions, predictive factors, pluripathology, polypharmacy

## Abstract

**Introduction:**

L’insuffisance cardiaque (IC) est un problème de santé en Espagne dont l’incidence croissante est en rapport avec le vieillissement de la population, et présente des taux de mortalité et de réadmissions hospitalières élevées. Evaluer les caractéristiques cliniques des patients souffrant d’IC qui entrent en médecine interne et les facteurs associés à la réadmission et la mortalité intrahospitalière.

**Méthodes:**

étude transversale, descriptive et rétrospective de révision des dossiers cliniques de patients ayant un diagnostic principal d’IC dans l’Ensemble Minimal de Base de Données (CMBD, *Conjunto Mínimo Básico de Datos*), qui abandonnèrent le Service de Médecine Interne de l’Hôpital Clinique San Carlos (HCSC) en 2014.

**Résultats:**

Cent quatre-vingt-dix-neuf (199) patients y furent inclus, âge moyen 82,7 ans et 61,8% de femmes; 85% présentaient une FEVG (Fraction d’Ejection du Ventricule Gauche) > 40%, avec un pro-BNP moyen de 9.101,3 pg/ml; 64,3% des patients présentèrent une fibrillation auriculaire permanente. 30,2% revinrent en < 30 jours avec une moyenne de réadmissions/an de 1,45(±0,86). 25% des patients moururent intra hospitalièrement pendant le suivi. Parmi les facteurs associés à la mortalité intrahospitalière l’âge avancé fut une variable associée OR 1,050 (1,002-1,101) (p = 0.04). Quant aux facteurs associés à la réadmission précoce, la polypharmacie (p = 0,024) ainsi que les critères de pluripathologie d’Ollero 4,974 (1,396-17,730) (p = 0,024) furent les plus importants. Les patients hospitalisés pour cause d’IC dans notre milieu sont des patients âgés et sous polymédication.

**Conclusion:**

Les patients hospitalisés pour insuffisance cardiaque sont âgés, présentant pluripathologie et polypharmacie. Son pronostic à court terme est associé à des taux élevés de réintégration et de mortalité à l'hôpital principalement pour ceux souffrant d’affection rénale et/ou neurologique.

## Abstract (en)

**Introduction:**

Heart failure (HF) is a health problem in Spain where the prevalence rate for this disease is correlated with aging. Heart failure-related mortality and hospital readmissions are high. The purpose of this study was to evaluate the clinical features of patients with HF hospitalized in the Department of Internal Medicine as well as factors associated with readmission and intra-hospital mortality.

**Methods:**

We conducted a cross-sectional, descriptive, and retrospective study based on the review of the clinical records of patients with primary diagnosis of HF in the Basic Minimum Set of Data (BMSD, Conjunto Mínimo Básico de Datos),who were discharged from the Department of Internal Medicine of the San Carlos Clinical Hospital (HCSC) in 2014.

**Results:**

The study involved 199 patients, with an average age of 82.7 years (61.8% were females); 85% of them had left ventricular ejection fraction (LVEF) > 40%, with an average pro-BNP of 9.101,3 pg/ml and 64.3% had ongoing atrial fibrillation. Thirty point two percent of patients were readmitted within 30 days, with an average rate of readmission/year of 1.45 (±0.86). Twenty five percent of patients died during the follow-up period in hospital. Among factors associated with intra-hospital mortality, older age was an associated variable (OR 1,050)(1,002-1,101) (p = 0.04). The most important factors associated with early readmission were polypharmacy (p = 0.024) as well as pluripathology based on Ollero criteria 4,974 (1,396-17,730) (p = 0.024). Patients hospitalized for HF in our Department are elderly patients treated with polymedication.

**Conclusion:**

Patients hospitalized for cardiac insufficiency are older and are characterized by pluripathology and polypharmacy. Short-term prognosis is associated with high rates of readmission and mortality in hospitalmainly for patients suffering from kidney disease and/or neurological disorders.

## Introduction

L’insuffisance cardiaque (IC) est un des problèmes de santé publique et de consommation de ressources sanitaires majeurs dans les sociétés occidentales. Son taux d’incidence croissant, en rapport avec le vieillissement de la population et peut-être avec la survie des syndromes coronaires aigus, a fait qu’elle soit qualifiée de véritable épidémie, en présentant des taux de mortalité et de réadmissions élevés malgré les progrès réalisés dans son traitement [1-3]. Dans les pays développés de 1 à 2% de la population environ souffrent d’IC, en pouvant dépasser 10% dans la population âgée de plus de 70 ans [2, 3]. Avant 1990, de 60 à 70% des patients mouraient lors des 5 ans suivant le diagnostic ou la première admission hospitalière. Le traitement efficace de l’IC a amélioré ces résultats, avec une réduction relative de l’hospitalisation de 30 à 50% lors des dernières années et des diminutions plus faibles mais importantes dans la mortalité [1, 4]. L’objectif de la gestion de l’IC est de fournir un système continu d’attention, en englobant la communauté et l’hôpital, afin de s’assurer que la manipulation de chaque patient soit optimale et éviter la réadmission [4, 5]. Notre étude a pour but de déterminer les caractéristiques cliniques des patients hospitalisés avec un diagnostic d’IC, les facteurs de prédiction associés à la réadmission et la mortalité intrahospitalière.

## Méthodes

Nous avons réalisé une étude transversale, descriptive et rétrospective de révision des dossiers cliniques de patients diagnostiqués à la sortie avec le CMBD de l’HCSC avec le code principal d’IC (code 428.0 de la CIE9). Nous avons inclus un total de 199 patients avec un diagnostic principal d’insuffisance cardiaque par CMBD, qui ont abandonné le Service de Médecine Interne de l’Hôpital Clinique San Carlos (HCSC) lors de l’année 2014 (du 1 janvier au 31 décembre 2014). Les variables de l’étude à analyser furent: le sexe, l’âge, les symptômes à l’admission et le facteur de déclenchement, les journées d’hospitalisation, le nombre de réadmissions, le nombre de jours jusqu’à la visite par le Médecin d’Attention Primaire (MAP) après la sortie, la situation fonctionnelle, diabète, hypertension artérielle (HTA), dyslipidémie, tabagisme, échocardiographie lors de la dernière année, fraction d’éjection du ventricule gauche (FEVG), présence de valvulopathies, hypertension pulmonaire (HTP), porteur de stimulateurs cardiaques ou de prothèses valvulaires, diagnostic de fibrillation auriculaire (FA), nécessité de transfusion sanguine pendant l’admission et nombre d’unités. Nous avons calculé l’indice de comorbilité de Charlson [6] abrégé et évalué le degré de pluripathologie à l’aide de l’échelle de Manuel Ollero [7]. Analytiquement, nous avons recueilli les variables suivantes à l’admission et à la sortie: les valeurs de pro-BNP, protéine C réactive (PCR), hémoglobine, ADE, ferritine, sodium, potassium, créatinine, filtrage glomérulaire (MDRD) et urée et uniquement à l’admission: phosphore, acide urique, billirubine et albumine.

Les autres variables recueillies furent: le nombre de médicaments consommés avant l’admission/après la sortie hospitalière, la consommation de médicaments associés au traitement de l’IC avant l’admission/après la sortie hospitalière (furosémide, spironolactone, thiazides, bêtabloquants, IECAs, ARA-II, inhibiteurs de canaux calciques, digoxine), consommation d’AINS, antiagrégants et anticoagulants. Ces variables furent obtenues à l’aide du Programme Patient, Horus et Eolhis. Enfin nous avons évalué la fréquence de réadmission en moins de 30 jours, la mortabilité intrahospitalière et le nombre de réadmissions globales pendant l’année 2014. Nous avons réalisé une analyse statistique descriptive avec les variables recueillies et une analyse bivariée pour comparer les caractéristiques des patients qui revinrent ou moururent par rapport à ceux qui ne le firent pas. Nous avons utilisé les essais statistiques T-student pour les variables quantitatives et Chi carré pour les qualitatives avec une signification statistique p < 0.05. Enfin, nous avons réalisé une analyse multivariée de régression logistique avec les variables cliniques qui furent associées à la réadmission ou à la mortalité intrahospitalière avec les variables d’âge, sexe, comorbidités associées et d’autres variables avec lesquelles nous avons obtenu une signification statistique (p < 0.05). L’analyse fut menée à bien en utilisant le programme SPSS version 21.

## Résultats

Les caractéristiques des patients avec un diagnostic principal d’IC sont décrites dans le Tableau 1. Entre les 199 patients inclus dans l’étude, la moyenne d’âge fut de 82,7 ans, avec 61,8% de femmes. Le degré de DABVD (dépendance pour les activités de la vie quotidienne) fut de 51,8%. Parmi les FRCV, 39,7% avaient DM, HTA (87,9%), dyslipidémie (64,8%) et des antécédents de tabagisme (29,6%). 85% avaient une FEVG supérieure à 40% et la FA était présente dans 64,35 des cas. La cardiopathie ischémique fut constatée chez 27,2% des sujets. Les valvulopathies de degré modéré ou grave les plus fréquentes furent: la sténose aortique pour 12,7%, l’insuffisance mitrale pour 21,5% et l’insuffisance tricuspidienne pour 19,2%. 56,3% souffraient une HTP modérée ou grave et 13,1% portaient des valves prothésiques ou des stimulateurs cardiaques.

**Tableau 1:** Analyse descriptive des 199 patients diagnostiqués d’IC à la sortie par CMBD

VARIABLES	N/total	Fréquence	Moyenne	σ
Âge	199/199		82,7	±7,7
Sexe	199/199	F (61,8%)		
		H (38,2%)		
Situation fonctionnelle	199/199	IABVQ (48,2%)		
		DABVQ (51,8%)		
Hospitalisations/an	199/199		1,45	±0,86
Séjour moyen hospitalisation (jours)			10,9	±8,1
Réadmis <30 jours	199/199	30,2%		
Décès intrahospitalier*		25,1%		
Visite chez le MAP <10 jours	199/199	OUI 48,2%,	6,2	±6,4
		NON 14,1%,		
		NS 37,7%		
**Échocardio dernière année**	**173/199**	**86,9%**		
FEVG<40 %		15%		
Dilatation AD modérée/grave		44%		
Diamètre AG (cm)			4,60	±0,82
DVITD (cm)			4,82	±0,8
DVITS (cm)			3,20	±0,9
Sténose AO modérée/grave		12,7%		
I. Mitrale modérée/grave		21,5%		
I. Tricuspide modérée/grave		19,2%		
HTP modérée/grave (>45mmHg)		56,3%		
Porteur de prothèse valvulaire/stimulateur cardiaque				
adorprótesis valvular / marcapasos		13,1% / 13,1%		
Fibrillation auriculaire	199/199	64,3%		
Cardiopathie ischémique	199/199	27,6%		
**Données analytiques**				
Pro-BNP (pg/ml)	151	84%	9101,30	±11793,14
PCR admission (mg/dl)	189		3,51	±4,47
PCR sortie (mg/dl)	115		1,56	±1,96
Hémoglobine admission (g/dl)	199		11,76	±2,10
Hémoglobine sortie (g/dl)	182		11,87	±1,83
ADE admission (%)	194		17,78	±3,20
ADE sortie (%)	163		17,60	±3,36
Na+ admission (mEq/L)	198		137,40	±5,06
Na+ sortie (mEq/L)	180		138,27	±3,81
K+ admission (mEq/L)	192		4,56	±0,72
K+ sortie (mEq/L)	166		4,15	±0,50
Phosphore (mg/dl)	126		3,63	±0,77
Acide urique (mg/dl)	102		7,82	±2,5
Bilirrubine (mg/dl)	132		0,80	±0,62
Albumine (g/dl)	124		3,39	±0,46
Créatinine admission (mg/dl)	199		1,38	±0,70
Créatinine sortie (mg/dl)	178		1,28	±0,60
MDRD admission (mL/min/1,73 m2)	198		51,53)	±24,84
MDRD sortie (mL/min/1,73 m2)	176		53,19	±22,55
Urée admission (mg/dl)	194		73,44)	±45,74
Urée sortie (mg/dl)	169		74,40	±41,50

FEVI: fraction d’éjection du ventricule gauche, AD : auricule droite, AI : auricule gauche, mod/sev : modéré/grave, I: insuffisance, DVGTD: diámetro ventrículo izquierdo telediastólico, DVGTDS: diamètre du ventricule gauche télésystolique, HTP: hypertension pulmonaire.

**Figure 1:**
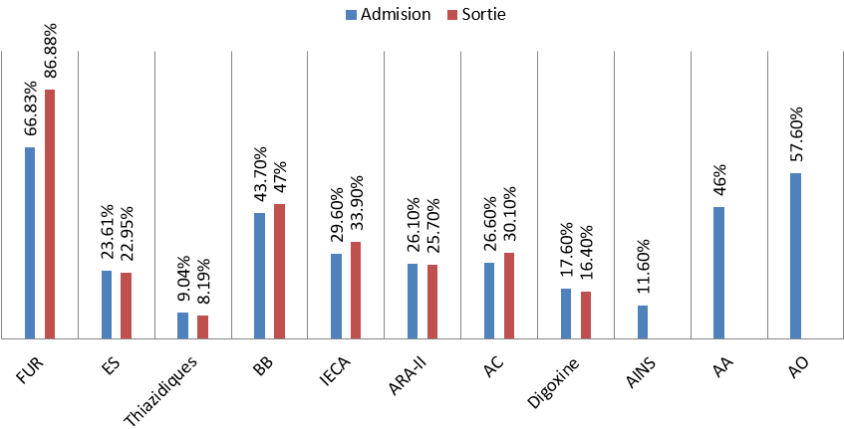
Traitement pharmacologique à l’admission et à la sortie (FUR: furosémide; ES: spironolactone; BB: bêta-bloquants, IECA: inhibiteurs de l’enzyme de conversion de l’angiotensine; ARA-II: antagonistes du récepteur de l’angiotensine-II; AC: antagonistes du calcium; AINs: Anti-inflammatoires non stéroïdiens; AA: anti-agréggants; AO: anticoagulants oraux)

Parmi les symptômes à l’admission, 85,9% des patients présentèrent une dyspnée, 39,2% des oedèmes périphériques, 33,2% une infection respiratoire aiguë et 8% une douleur thoracique. La classification fonctionnelle basale (NYHA) ne put s’y refléter étant donné qu’elle ne fut recueillie dans le dossier clinique que dans très peu de cas. Nous avons évalué si les patients respectaient les critères de Framingham pour IC à l’admission où ils étaient présents dans 73,2% La comorbidité des patients fut évaluée à l’aide de l’indice de Charlson abrégé (0-10) avec une ponctuation moyenne de 2,57 (±1,24). À l’aide des critères d’Ollero pour l’évaluation de prévalence des patients pluripathologiques (2 catégories ou plus) nous avons estimé que 80,9% des patients présentaient une pluripathologie. À l’admission nous avons quantifié le pro-BNP de 84% des patients avec des valeurs moyennes de 9101,3 pg/ml (±11793,14). La valeur moyenne d’hémoglobine du total de l’échantillon fut de 11,76 g/dl (±2,10), étant inférieure à 10 mg/dl chez 19,6% des patients, et celle de créatinine de 1,38 mg/dl (±0,70), 65,2% étant classés comme IRC état III et 20,7% comme état IV. Avant l’admission les patients prenaient 9±3 médicaments/jour et 9,5±3 à la sortie. 57,6% prenaient des anticoagulants oraux (AO), 46% des antiagrégants et 11,6% seulement prenaient des AINS. À l’admission, 29,6% prenaient des IECAS, et 33,9% à la sortie et 43,7% prenaient des bêta-bloquants et 47% le faisaient à la sortie. Nous avons recueilli les patients qui prenaient des diurétiques, le pourcentage de prescription de furosémides augmenta de 20% à la sortie, tandis qu’il n’y eut pas de variations importantes dans la prescription de spironolactone et de thiazides (Figure 1).

La moyenne de réadmissions par an fut de 1,45 (±0,86), 30,2% des patients revenant en < 30 jours avec un séjour hospitalier moyen de 10,9 jours (±8,1), et était supérieure chez les patients qui ne moururent pas (p = 0,039). Les patients qui reviennent en < 30 jours consomment un plus grand nombre de médicaments (10,17±3,28; p = 0,001). D’autre part, nous observons qu’il y a une association de réadmissions majeure chez les patients souffrant de pluripathologie qui respectent les critères B (maladie rénale) (p = 0,005) et E (neurologique) (p = 0,045). La consommation d’IECAs est supérieure chez les patients qui revenaient en < 30 jours (p = 0.02) (Tableau 2 et Tableau 3). 25,1% moururent en milieu hospitalier pendant la période de suivi; ces patients avaient un âge moyen supérieur (p = 0,035) (Tableau 4). Parmi ceux qui ne moururent pas, 48,2% allèrent chez leur MAP en moins de 10 jours (6,2±6,4). Les patients qui n’étaient pas sous anticoagulants au préalable moururent plus (p = 0,038). Un échocardiogramme avait été réalisé à 86,9% des sujets pendant l’an dernier. Le fait de l’avoir est associé à un nombre de décès inférieur (p = 0,030). Enfin, nous avons réalisé une analyse de régression logistique en incluant la variable d’âge, sexe et comorbilité de Charlson, l’âge étant le facteur le plus associé à la mortalité avec une OR 1,050 (1,002-1,101) (p = 0,04). Quant aux facteurs associés à la réadmission précoce, la polypharmacie (p = 0,024) ainsi que les critères de pluripathologie d’Ollero 4,974 (1,396-17,730) (p = 0,024) furent les critères indépendants. Les IC de 95% sont reflétées dans les Tableau 5 et Tableau 6.

**Tableau 2:** Analyse bivariée des variables analysées chez les 199 patients souffrant d’ICC et la réadmission précoce (30 jours); caractéristiques, données échocardiographiques et valeurs analytiques

Variables (n) / Moyenne / %	Réadmission en <30 jours	Non réadmission en < 30 jours	P<0.05
	N = 64	N = 135	
**Caractéristiques**			
**Âge**	(64) 81,80±9,03	(135) 83,51±6,97	0,143
**Sexe (femme)**	(36) 56,3%	(87) 64,4%	0,266
**Nº admissions/années**	(64) 1,72±1,16	(135) 1,32±0,64	***0,002***
**Nº jours d’hospitalisation**	(64) 11,84±8,35	(135) 10,54±7,97	0,290
**Décès**	(16) 25%	(34) 25,2%	0,978
**Réadmission**	(28) 43,8%	(32) 23,7%	***0,004***
**Situation fonctionnelle**	(36) 56,3%	(67) 49,6%	0,383
**Infection respiratoire**	(20) 31,3%	(46) 34,1%	0,693
**Diabètes Mellitus**	(26) 40,6%	(53) 39,3%	0,854
**HTA**	(55) 85,9%	(120) 88,9%	0,550
**Dyslipidémie**	(38) 59,4%	(91) 67,4%	0,268
**Tabagisme**	(23) 35,9%	(36) 26,7%	0,181
**Données échocardiographiques**			
**Échocardiogramme dernière année**	(56) 87,5%	(117) 86,7%	0,871
**FEVG < 40%**	(8) 14,3%	(18) 15,4%	0,850
**AD dilatation mod/grave**	(21) 53,8%	(34) 39,5%	0,135
**AI diamètre (cm)**	(49) 4,55±0,71	(107) 4,63±0,87	0,589
**DVGTD**	(50) 4,80±0,78	(98) 4,83±0,82	0,828
**DVITS**	(33) 3,04±0,64	(79) 3,27±0,99	0,212
**E. aortique mod/grav**	(6) 10,7%	(16) 13,7%	0,584
**I. mitrale mod/grav**	(11) 19,6%	(26) 22,4%	0,679
**I. tricuspide mod/grav**	(10) 17,9%	(23) 19,8%	0,758
**http**	(31) 55,4%	(67) 56,8%	0,860
**Cardiopathie ischémique**	(16) 25%	(39) 28,9%	0,567
**Prothèse valvulaire**	(9) 14,1%	(17) 12,6%	0,774
**FA**	(43) 67,2%	(85) 63%	0,561
**Stimulateur cardiaque**	(12) 18,8%	(14) 10,4%	0,101
**Valeurs analytiques**			
**Valeur pro-BNP** (pg/ml)	(47) 11624,36±16301,51	(104) 9345,69±17364,34	0,448
**Pro-BNP>1500** (pg/ml)	(41) 87,2%	(86) 82,7%	0,480
**Hémoglobine admission**(g/dl)	(64) 11,72±2,44	(135) 11,78±1,93	0,837
**Hémoglobine sortie** (g/dl)	(58) 11,99±1,85	(124) 11,82±1,82	0,552
**Sodium admission** (mEq/L)	(64) 138,37±5,30	(134) 136,94±4,90	0,062
**Sodium sortie** (mEq/L)	(57) 138,58±4,25	(123) 138,13±3,60	0,464
**Potassium admission** (mEq/L)	(64) 4,39±0,70	(128) 4,65±0,73	***0,023***
**Potassium sortie** (mEq/L)	(55) 4,07±0,53	(111) 4,19±0,50	0,142
**Acide urique** (mg/dl)	(36) 8,19±2,94	(66) 7,62±2,23	0,272
**Albumine** (g/dl)	(41) 3,31±0,42	(83) 3,43±0,48	0,175
**Urée admission** (mg/dl)	(64) 73,95±40,02	(130) 73,20±48,46	0,914
**Urée sortie** (mg/dl)	(54) 69,11±38,17	(115) 76,90±42,92	0,257
**Créatinine admission** (mg/dl)	(64) 1,36±0,74	(135) 1,40±0,68	0,689
**Créatinine sortie** (mg/dl)	(56) 1,28±0,76	(122) 1,29±0,55	0,918
**MDRD admission(**mL/min/1,73m2)	(64) 55,67±27,81	(134) 49,55±23,14	0,105
**MDRD sortie (**mL/min/1,73 m2)	(55) 58,27±27,51	(121) 50,88±19,59	***0,044***

FRCV: facteurs de risque cardiovasculaire, (*): pendant la période de suivi, FEVG: fraction d’éjection du ventricule gauche, AD: auricule droite, AI: auricule gauche, mod/sev : modéré/grave, I: insuffisance, DVGTD: diamètre ventricule gauche télésystolique, DVITDS: diamètre du ventricule gauche télésystolique, HTP: hypertension pulmonaire.

**Tableau 3:** Analyse bivariée des variables analysées chez les 199 patients souffrant d’ICC et la réadmission précoce (30 jours); médicaments et échelles

Variables (n) / Moyenne/ %	Réadmission en <30 jours	Non réadmission en < 30 jours	P<0.05
	N = 64	N = 135	
**Médicaments**			
**Nº médicaments préalables**	(64) 10,17±3,28	(135) 8,50±3,20	***0,001***
**Nº médicaments sortie**	(59) 10,68±3,02	(124) 8,98±3,23	***0,001***
**Furosémide entrée (mg)**	(43) 51,63±29,67	(90) 61,56±30,16	0,077
**Furosémide sortie (mg)**	(51) 61,57±33,67	(108) 62,41±32,32	0,880
**Spironolactone entrée**	(13) 29,8±21,37	(34) 27,94±16,30	0,749
**Spironolactone sortie**	(11) 34,09±23,11	(31) 28,63±20,20	0,462
**Échelles**			
**Respectent les critères de Framingham pour IC**	(49) 76,6%	(96) 71,6%	0,465
**Indice de Charlson**	(64) 2,78±1,24	(135) 2,47±1,24	0,105
**Ollero >/= 2**	(60) 93,8%	(101) 74,8%	***0,002***

**Tableau 4:** Analyse bivariée des variables analysées chez les 199 patients souffrant d’ICC et la mortalité intrahospitalière

Variables (n) / moyenne %	Décès	Non Décès	P<0.05
	N = 50	N = 149	
**Caractéristiques**			
**Âge**	(50) 84,80±6,71	(149) 82,34±7,95	***0,035***
**Sexe**	(32) 64%	(91) 61,1%	0,712
**Nº entrées/an**	(50) 1,62±1,18	(149) 1,39±0,72	0,102
**Nº réadmissions < 30 jours/an**	(50) 0,38±0,60	(149) 0,36±0,55	0,791
**Nº jours d’hospitalisation**	(50) 9,16±6,47	(149) 11,56±8,51	***0,039***
**Réadmis**	(18) 36%	(42) 28,2%	0,298
**Situation fonctionnelle DAVBD**	(31) 62%	(72) 48,3%	0,094
**Infection respiratoire**	(18) 36%	(48) 32,2%	0,623
**Diabètes mellitus**	(16) 32%	(63) 42,3%	0,199
**HTA**	(39) 78%	(136) 91,3%	***0,013***
**Dyslipidémie**	(31) 62%	(98) 65,8%	0,629
**Tabagisme**	(16) 32%	(43) 28,9%	0,674
**Données échocardiographiques**			
**Échocardiogramme dernière année**	(39) 78%	(134) 89,9%	***0,030***
**FEVG<40%**	(6) 15,4%	(20) 14,9%	0,944
**AD dilatation mod/grav**	(13) 44,8%	(42) 43,8%	0,918
**AI diamètre (cm)**	(37) 4,58±0,10	(119) 4,61±0,77	0,825
**DVITD (cm)**	(35) 4,84±0,94	(113) 4,82±0,76	0,901
**DVITS (cm)**	(24) 3,23±0,94	(88) 3,20±0,90	0,860
**E. aortique mod/grav**	(7) 31,8%	(15) 68,2%	0,300
**I. mitrale mod/grav**	(9) 23,1%	(28) 21,1%	0,787
**I. tricuspide mod/grav**	(6) 15,4%	(27) 20,3%	0,493
**HTP (PSAP>45 mmHg)**	(21) 52,5%	(77) 57,5%	0,579
**Cardiopathie ischémique**	(16) 32%	(39) 26,2%	0,425
**Prothèse valvulaire**	(7) 14%	(19) 12,8%	0,821
**FA**	(29) 58%	(99) 66,4%	0,281
**Stimulateur cardiaque**	(7) 14%	(19) 12,8%	0,821
**Valeurs analytiques**			
**Valeur pro-BNP** (pg/ml)	(33) 10341,94±10826,03	(118) 9974,69±18419,31	0,913
**Pro-BNP>1500** (pg/ml)	(28) 84,8%	(99) 83,9%	0,895
**Hémoglobine** (g/dl)	(50) 11,51±1,99	(149) 11,84±2,14	0,325
**Sodium** (mEq/L)	(50) 137,14±5,07	(148) 137,49±5,07	0,671
**Potassium** (mEq/L)	(49) 4,77±0,92	(143) 4,49±0,64	***0,019***
**Albumine**(g/dl)	(29) 3,28±0,56	(95) 3,43±0,42	0,121
**Urée** (mg/dl)	(50) 79,40±39,37	(144) 71,38±47,71	0,244
**Créatinine** (mg/dl)	(50) 1,57±0,87	(149) 1,32±0,62	***0,027***
**MDRD** (ml/min/1,73 m2)	(49) 47,59±29,84	(149) 52,83±22,93	0,202
**Médicaments**			
**Nº médicaments préalables**	(50) 8,74±3,46	(149) 9,13±3,267	0,468
**Furosémide**	(38) 57,37±31,94	(95) 58,74±29,72	0,815
**Spironolactone**	(15) 25,83±7,42	(32) 29,68±20,75	0,491
**Échelles**			
**Respectent les Critères de Framingham pour l’ IC**	(39) 79,6%	(106) 71,1%	0,246
**Indice de Charlson**	(50) 2,74±1,27	(149) 2,52±1,24	0,275
**Ollero >/= 2**	(42) 84%	(119) 79,9%	0,520

FRCV: facteurs de risque cardiovasculaire, (*): pendant la période de suivi, FEVG: fraction d’éjection du ventricule gauche, AD: auricule droite, AI: auricule gauche, mod/sev: modéré/grave, I: insuffisance, DVGTD: diamètre ventricule gauche télésystolique, DVITDS: diamètre du ventricule gauche télésystolique, HTP: hypertension pulmonaire.

**Tableau 5:** Analyse multivariée en décès

	95% IC	Valeur P
**Âge**	1,050 (1,002-1,101)	0,04
**Sexe**	0,865 (0,426-1,756)	0,68
**Admissions/An**	1,328 (0,918-1,919)	0,13
**CHARLSON**	1,159 (0,890-1,510)	0,27

**Tableau 6:** Analyse multivariée en réadmission

	95% IC	Valeur P
**Âge**	0,975 (0,933- 1,018)	0,249
**Sexe**	1,228 (0,617-2,445)	0,558
**Ollero**	4,974 (1,396-17,730)	0,013
**Nº médicaments sortie**	1,137 (1,017-1,272)	0,024

## Discussion

Les principales données dérivées de cette étude nous permettent d’observer, en premier lieu, que la population hospitalisée pour cause d’IC dans notre milieu est très âgée car plus de la moitié des patients a plus de 84 ans. En comparaison avec les articles revus, la moyenne d’âge était inférieure à 80 ans dans la plupart des publications nationales [1, 8-11] et aux environs de 65 ans dans les publications internationales [12-15]. La distribution par sexes ne diffère pas de celle d’autres études. Nous détectons une prévalence de comorbidité élevée et de même que la majorité des études revues, nous observons que les principales maladies associées sont: HTA, dyslipidémie, DM, FA et cardiopathie ischémique. Le taux de mortalité intrahospitalière que nous avons trouvé est élevé si nous le comparons avec d’autres séries nationales [1, 10]. Dans notre étude le grand âge est fondamentalement associé à la mortalité. Ceci peut être dû à ce que la mortalité intrahospitalière est fondamentalement associée aux individus ayant un âge moyen majeur qui sont hospitalisés dans des conditions de santé pires. Leur séjour fut plus court que celui de ceux qui survécurent. Cette donnée est opposée à d’autres études, où la mortalité fut mise en rapport avec un séjour hospitalier plus long [8]. Dans notre série l’antécédent d’HTA, l’hyperkaliémie, des valeurs de créatinine supérieures [1, 8] et la non anticoagulation furent également mis en rapport avec une augmentation de la mortalité.

Enfin un autre facteur associé à la mortalité est le fait de ne pas avoir réalisé un échocardiogramme lors de l’an dernier: c’est pourquoi, de même que Galofré *et al.* [10] nous considérons que la réalisation de l’échocardiogramme est fondamentale pour déterminer la fonction ventriculaire, outre d’avoir des implications thérapeutiques. Cependant, nous n’avons pas pu obtenir de signification statistique dans l’analyse multivariée pour ces variables. Bien qu’il s’agisse d’une population chez laquelle 85% ont une fonction systolique conservée, dans notre étude il n’y a pas de données échocardiographiques qui apporteraient une valeur pronostique, mais le simple fait de le réaliser. L’association entre la mortalité et la non anticoagulation est une donnée à souligner. Les patients qui présentaient une comorbidité majeure ne prenaient pas d’anticoagulants et moururent plus que ceux auxquels ce traitement n’était pas prescrit. C’est une réalité que plus la comorbidité est importante plus le risque thromboembolique est grand et majeur le bénéfice du traitement avec des anticoagulants oraux [16, 17], mais en raison de la faible espérance de vie et de la situation de gravité il est possible qu’ils n’aient pas reçu le traitement avec des anticoagulants en évaluant la situation de risque-bénéfice.

Dans notre groupe, il est important de souligner l’existence d’un degré élevé de polypharmacie. Par polypharmacie on entend le fait de prendre plus de médicaments que ceux cliniquement appropriés, le nombre le plus étendu étant de 5 médicaments utilisés de manière chronique [18]. De même que Picker *et al.* [12] nous trouvons un rapport statistiquement important entre une consommation majeure de médicaments à la sortie et une augmentation du nombre de réadmissions. Ils concluent que l’introduction du degré de polypharmacie dans les échelles de stratification du risque de ces patients pourrait être utile. Cette découverte peut paraître nouvelle étant donné que dans notre milieu nous n’avons pas évalué autant la variable de polypharmacie. De plus dans leur étude, ils suggèrent que les patients polymédicamentés présentent une complexité et une gravité majeures et ont une plus grande possibilité d’un mauvais respect thérapeutique. Dans notre recherche il ne nous futpas possible d’évaluer le degré d’adhérence thérapeutique, mais l’âge si avancé de l’échantillon et la consommation élevée de médicaments pourraient suggérer ce facteur comme éventuel déclencheur d’un nouvel épisode.

La prévalence élevée de pluripathologie attire l’attention dans l’échantillon étudié. La définition fonctionnelle de patient pluripathologique conçue par Ollero *et al.* [7] appliquée de manière rétrospective et à partir uniquement de l’information fournie par le CMBD, est un bon outil pour différencier ce groupe de patients Dans l’étude réalisée par Fernández Miera [11], 40,3% uniquement des patients admis en Médecine Interne respectaient ces critères, sans spécifier les catégories étaient les plus prévalentes contrairement à notre étude dans laquelle nous mettions en évidence une incidence majeure de réadmission chez les patients souffrant d’affection rénale et d’une maladie neurologique associée à une maladie neurovasculaire et à la démence. Dans l’étude réalisée par Chamberlain *et al.* [19] il associe un facteur de risque majeur de réadmission à ceux les plus âgés et ayant une comorbidité plus importante de même que nous pouvons l’observer dans notre étude. Par contre, la prise de bêta-bloquants, diurétiques de l’anse et thiazides qui semble associé un risque majeur de réadmission, ne coïncide pas dans notre cas. De plus, la prise d’IECA, ARA-II et spironolactone sont des facteurs qui préviennent la réadmission et dont les résultats s’opposent dans la prise d’IECAS à ceux de notre étude, dans lesquels il existe une association importante avec la prise d’IECAS avant l’admission et la réadmission en moins de 30 jours.

Compte tenu de la prévalence élevée de réadmissions en moins de 30 jours après la sortie, il serait très utile de réaliser une échelle de stratification du risque qui permettrait de prédire les patients à haut risque. Selon Dunbar – Yaffe *et al.* [5] et Ruiz Romero [9] il est possible de réaliser 3 types d'interventions: la première serait d’identifier, pendant l’admission, les patients à haut risque, la seconde serait fondée sur des stratégies ambulatoires pour un meilleur contrôle après la sortie et enfin, développer un algorithme d’Urgence pour stratifier le risque de mortalité et pouvoir déterminer ainsi si l’admission est nécessaire ou s’il peut être manipulé de manière ambulatoire. De plus, dans leur étude, Padhukasahasram *et al.* [20] concluent l’importance d’inclure dans les échelles non seulement des valeurs cliniques comme il arrive dans la plupart mais également des facteurs socio-économiques et de qualité de vie. Enfin, compte tenu des découvertes réalisées dans notre recherche, il semble important d’établir des stratégies d’intervention d’assistance pour prévenir les réadmissions précoces pour cause d’insuffisance cardiaque en coordination avec une infirmerie spécialisée en IC, Cardiologie, Médecine Interne, Gériatrie et Attention Primaire. Finalement, compte tenu de l’âge avancé et de la pluripathologie des patients souffrant d’IC hospitalisés en Médecine Interne, dans notre milieu, il semble également très prioritaire de développer des stratégies d’attention au patient chronique complexe et terminal par le biais de programmes d’attention multidisciplinaire qui incluraient l’attention à domicile et les soins palliatifs.

### Limitations

Comme il s’agit d’une étude rétrospective basée sur le CMBD et sur l’accès à l’information clinique par le biais d’outils informatiques, il peut y avoir une probabilité majeure d’infracodification dans certaines variables cliniques. Par exemple, il ne fut pas possible d’évaluer la clase fonctionnelle (NYHA), le degré de dépendance fonctionnelle (Barthel), ou la situation socioéconomique des patients car ils ne figuraient pas dans les rapports de sortie ni dans le dossier clinique électronique. D’autre part, nous n’avons pas pu obtenir des informations s’il y avait un bon respect thérapeutique de la part des patients, car elles n’étaient pas recueillies dans le dossier clinique; tous ces facteurs étant ceux qui influencent la réadmission à court terme et la mortalité. Enfin, nous trouvons des limitations dans le groupe de patients inclus, patients âgés avec une pluripathologie élevée et dans des phases d’IC élevées, car ils appartiennent à un environnement géographique déterminé et, les conclusions obtenues dans ce travail ne peuvent pas être généralisées à tous les patients souffrant d’IC. Il serait nécessaire de réaliser des études avec un échantillon de population représentatif dans d’autres centres de la Communauté de Madrid ou au niveau national dans les services de Médecine Interne. Pour conclure, nous sommes conscients des limitations concernant le pouvoir statistique pour évaluer les facteurs de mortalité intrahospitalière et la réadmission avec un échantillon de 199 patients.

## Conclusion

Notre étude conclut que les patients hospitalisés pour cause d’IC dans notre milieu sont, pour la plupart, des patients âgés, de plus de 80 ans, avec une grande comorbidité et un degré élevé de polymédication (ils prennent plus de 5 médicaments par jour). La pluripathologie et la polypharmacie furent les facteurs les plus associés à la réadmission, principalement chez ceux souffrant d’affection rénale et/ou neurologique avec un taux élevé de réadmissions en moins de 30 jours après la sortie.

### Etat des connaissances actuelles sur le sujet

- L’incidence des hospitalisations due à l’insuffisance cardiaque aigue est en augmentation;
- L’insuffisance cardiaque affecte principalement le sujet âgé présentant plusieurs comobidités;
- La comorbidité est évaluée habituellement selon l’index de Charlson, celui-ci a été fait sur des femmes atteintes d’un cancer du sein.

### Contribution de notre étude à la connaissance

- Un nombre important des patients hospitalisés en Médecine Interne sont pluripathologiques, c'est-à-dire, ils ont deux ou plus de maladies chroniques ou incurables;
- La pluripathologie dans l’insuffisance cardiaque identifie un groupe de patients présentant une importante incapacité fonctionnelle, taux de réadmission et mortalité intrahospitalière;
- L’évaluation de la comorbidité chez le sujet âgé avec insuffisance cardiaque, sa situation fonctionnelle et cognitive, pourrait aider à identifier un groupe de patients relevant de soins palliatifs.

## Conflits d’intérêts

Les auteurs ne déclarent aucun conflit d'intérêts.
